# Palmistry Notes

**Published:** 1887-08-20

**Authors:** 


### Amusements for Convalescents.

### PALMISTRY NOTES.

By a Lady.

By the Way.

The art of cheirosophy or Palmistry is one of the oldest of the mystic arts; it is therefore ancient enough to have attractions for the modern mind. In the present pages, and in those which are to follow, I have put together some of the principal points connected with the study of Palmistry, hoping that they may not be without interest to the general reader. My first object has been to make the rudiments of the art as clear and concise as possible, so that those who have not hitherto paid any attention to the subject may, without difficulty, perfect themselves in the theory and practice of it. Patience in the acquirement of details, a power of observation, of deduction, and of insight, are all items which tend to a successful issue in the study of Palmistry. Earnestness in this, as in other matters, is a *sine qua non;* so to those who take up Palmistry as a matter of interest and amusement only—and it is from this aspect that I prefer to treat—I would say, "Adopt it truly, if only to be truly amusing, and you will have done a good thing." Do not take it up in parts, but as a whole; and in examining a hand look well at it, outwardly and inwardly, before essaying any "prophetic utterance." You will find it quite easy to arouse interest, for in spite of all that is said to the contrary, nothing is so interesting to individuals as the discussion of matters distinctly relating to themselves. Your primary object being one of amusement it is better to avoid speaking of any particularly bad sign to be found in a hand, such as a marked tendency to insanity or suicide, death by drowning, sudden death, etc. An examination can be made perfectly interesting without treading on tragic or dangerous ground. People often say: "I can understand there being something analogous in the type of a man's hand and his character; but no sensible person can believe that the course of his life is traced in his hand by the lines and marks found in it!" In reply, I would simply say, "Certain signs are discoverable in a hand, significant of certain things, and whether we believe those signs or not, it is interesting to hear what is to be said about them." My own experience leads me to the conviction that very odd coincidences between the lines in the hands and the events in the lives of individuals do exist. I offer no explanation of the fact; it is so, and that, to me, is enough.

Stage Accessories.

So much for preliminaries, now for a little practical illustration. First as to the mode of examination. Place yourself opposite your "subject," "grasp your nettle" by taking the hand gently but firmly in your own. The left hand is best for demonstration, although it is necessary to refer to the right hand for confirmation of a sign sometimes. A line or mark is not absolute in its significance unless it is found in both hands. Make your deductions from the back of the hand first; there the force of character is to be found. Next look at the texture, whether it be rough or smooth; examine the finger-tips (note if they are square, spatulate, or pointed), also the nails; the shape of the fingers: see if they are knotted, *i.e.*, with first and second joints developed, or if they are smooth in outline. Then note the colour of the hand; very white and firm it denotes selfishness, coldness, want of sympathy, excepting in things appertaining to itself. If of a wholesome redness, it gives cheerfulness, hopefulness, and quickness of temper. A perfectly smooth back to a hand shows, if it be very firm, egotism; a wrinkled condition of the skin, virulence; while the veins showing strongly is a sign of sympathy. A little hair on a man's hand denotes daring. Much hair gives a tendency to roving and inconstancy. Hair on the thumb, originality and skill; on a woman's hand hair shows cruelty, or in an otherwise good hand it may merely insure a tendency to "teaze."

From Back to Palm.

Having examined the back of the hand, turn it over, bending the fingers slightly forward to bring the lines in the palm into stronger relief. Hold it obliquely as regards the light; it will enable you to see the lines more distinctly.

*A palm, to be consistent* with the rest of the hand, should be in proportion with the fingers: neither too long nor too short. It should be firm, without being hard, and above all stiff; soft, without being limp.

*Short palm*, with long, knotty fingers, shows an over-anxious temperament, resulting in too great attention to detail at the expense of entirety; sentiment tending to fantasy.

*Long palm with short fingers*, obstinacy and tyranny, amounting to cruelty when thwarted.

*Narrow, thin palm*, timidity and delicacy; sometimes weakness of mind, shallowness, want of moral force.

*Hollow, deep palm*, constant struggle against difficulties, generally transmitted trials, such as poverty, hereditary disease, etc. If the line of fate be strong and straight, the phalange of will on the thumb powerful, the evil significance will be mitigated; and, with other good signs, the character of the individual becoming strengthened by successfully combating the difficulties as they arise, he will ultimately rise triumphant over all obstacles, and attain eminence through this means

(*To be continued.*)

